# A Case of Urethritis from Constricted Meatus

**Published:** 1877-02

**Authors:** R. W. Westmoreland

**Affiliations:** Atlanta, Ga.


### A CASE OF URETHRITIS FROM CONSTRICTED MEATUS.

By R. W. WESTMORELAND, M.D., Atlanta, Ga.

Mr. ——, æt. 21 years, consulted me in regard to a case of supposed gonorrhœa. The history of the case, as obtained from him, was that a month previously he noticed a discharge, and experienced some burning on urinating. He used an injection advised by some friend, which increased the difficulty, so that when he decided to undergo regular treatment, the urethra, for its whole extent, was found to be intensely inflamed. Besides several warty excrescences, the meatus was found to be diminished in size by cicatricial tissue at its inferior commissure. This latter condition was attributed to an injury received a year or two before, a result of rupture of the frenum.

A solution of nitrate of silver, gr. j to ℥j of water, was prescribed, but could not be borne, so great was the inflammation. Tinct. iodine was applied along the course of the urethra on the inferior surface, while cubebs and spirits nitre were given *per stoma.* Occasionally during this treatment attempts were made to use the injection, but always with the same result as at first.

During this time the discharge was not at all extensive; and so, with a view of obtunding the hyperesthetical nerves, an attempt was made to pass a steel sound. The instrument occasioned intense pain, along its course to the prostate, when it stopped, and the patient would permit it to be pressed no further. The urethra was involved in its whole extent, and the prostate was considerably enlarged and irritable; the patient being unable to retain his urine but a short time, and having to urinate three or four times oftener than natural. Incision of the meatus was determined on, and performed with almost immediate benefit. The injection could be used, the sound could be passed with comparative comfort, and the prostatitis was considerably ameliorated. Owing to neglect in keeping the pledget of lint in the incised meatus, the cut edges united, and a renewal of the former difficulties came on. Again, however, the meatus was opened, with the same marked benefit.

This was not the only trouble that resulted, as I suppose, from the contracted meatus, in the case referred to. A slight stricture just behind the meatus was found to exist, giving constant trouble, after the general irritability and inflammation, before alluded to, had been allayed. Now, when it is known that contracted meatus will give to the whole urethral mucous membrane, not only excessive sensibility, but actual irritation and inflammation, there can be no great surprise at finding a stricture, as the direct result of such state of the canal. In my case, after the general hyperesthesia had subsided, and there existed no longer any exciting cause in contracted meatus, this worse trouble of stricture had to be remedied before complete comfort could be restored to the patient.

For this purpose gradual dilatation was practiced for some weeks, with no decided and permanent enlargement of the contracted portion. Further attempts to relieve the difficulty without more violent means seemed useless, and rupture of the contracted part by the divulsor was determined upon. No considerable shock resulted from full enlargement by this means, nor any decided inflammatory action. The occasional introduction of dilating bougies, to prevent contraction of the canal, in the healing of ruptured tissues, completed the cure, and gave perfect relief from all unpleasant symptoms.

